# A Biological OR(XNOR) Logic Gate Couples Carbon Source and Transgene Expression Switching in a *Komagataella phaffii* (*Pichia pastoris*) Strain Co-producing Process-Enhancing Lipase and a Virus-like Particle (VLP) Vaccine

**DOI:** 10.1021/acssynbio.2c00342

**Published:** 2023-02-27

**Authors:** Sushobhan Bandyopadhyay, Vasos Pavlika, Daniel G. Bracewell, Darren N. Nesbeth

**Affiliations:** Department of Biochemical Engineering, University College London, Bernard Katz Building, London WC1E 6BT, United Kingdom

**Keywords:** Triacylglycerol lipase, logic gate, virus-like particle, vaccine, bioreactor, carbon source

## Abstract

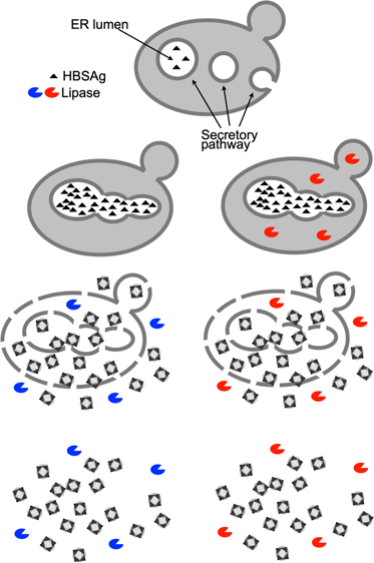

We constructed a three-input biological logic gate: S OR (G XNOR M), where S is sorbitol, G is glycerol, and M is methanol, to optimize co-expression of two transgenes in *Komagataella phaffii* using batch-mode carbon source switching (CSS). *K. phaffii* was engineered to harbor transgenes encoding a *Candida rugosa* triacylglycerol lipase, which can enhance downstream processing by removing host cell lipids from homogenates, and the hepatitis B virus surface antigen (HBsAg), a protein that self-assembles into a virus-like particle (VLP) vaccine. Using the native alcohol oxidase 1 (P_AOX1_) and enolase 1 (P_ENO1_) promoters to direct VLP vaccine and lipase expression, respectively, successfully provided an OR(XNOR) gate function with double-repression as the output. This logic gate functionality enabled use of CSS to ensure that approximately 80% of total VLP yield was accumulated before cells were burdened with lipase expression in 250 mL DasGip bioreactor cultivation.

Biological logic gates have been constructed and verified in many different organisms,^1^ including microbes and animal cells, and are now beginning to be deployed in biotechnology host cells in challenging industrial settings. *Komagataella phaffii* is used widely for production of complex biotherapeutics and vaccines, as a whole cell biocatalyst, and for production of recombinant enzymes.^2^*K. phaffii* is an established industrial host cell for production of vaccines in the “virus like particle” (VLP) format, whereby a single antigenic protein is selected for use as a vaccine based on its ability to self-assemble into capsid-like structures which are highly stable but have zero capacity for viral function. For example, *K. phaffii* is used for production of the Glaxo-SmithKline ENGERIX B vaccine which comprises self-assembled VLPs formed by the hepatitis B virus surface antigen (HBsAg) protein.^3^

VLP release from yeast cells requires total cell disruption which co-releases high levels of cell-derived lipids which can compromise the downstream filtration steps used to purify the VLPs at scale.^4^ We previously showed that treatment of *K. phaffii*-derived HBsAg VLP process streams with exogenous *Candida rugosa* triacylglycerol Lip3 lipase (Figure 1A, steps 1–4) improved the performance of downstream filtration steps.^5^ Despite these benefits, adding prepurified exogenous proteins to an industrial process always exposes that process to the risk of batch variation from suppliers and to additional costs.

**Figure 1:**
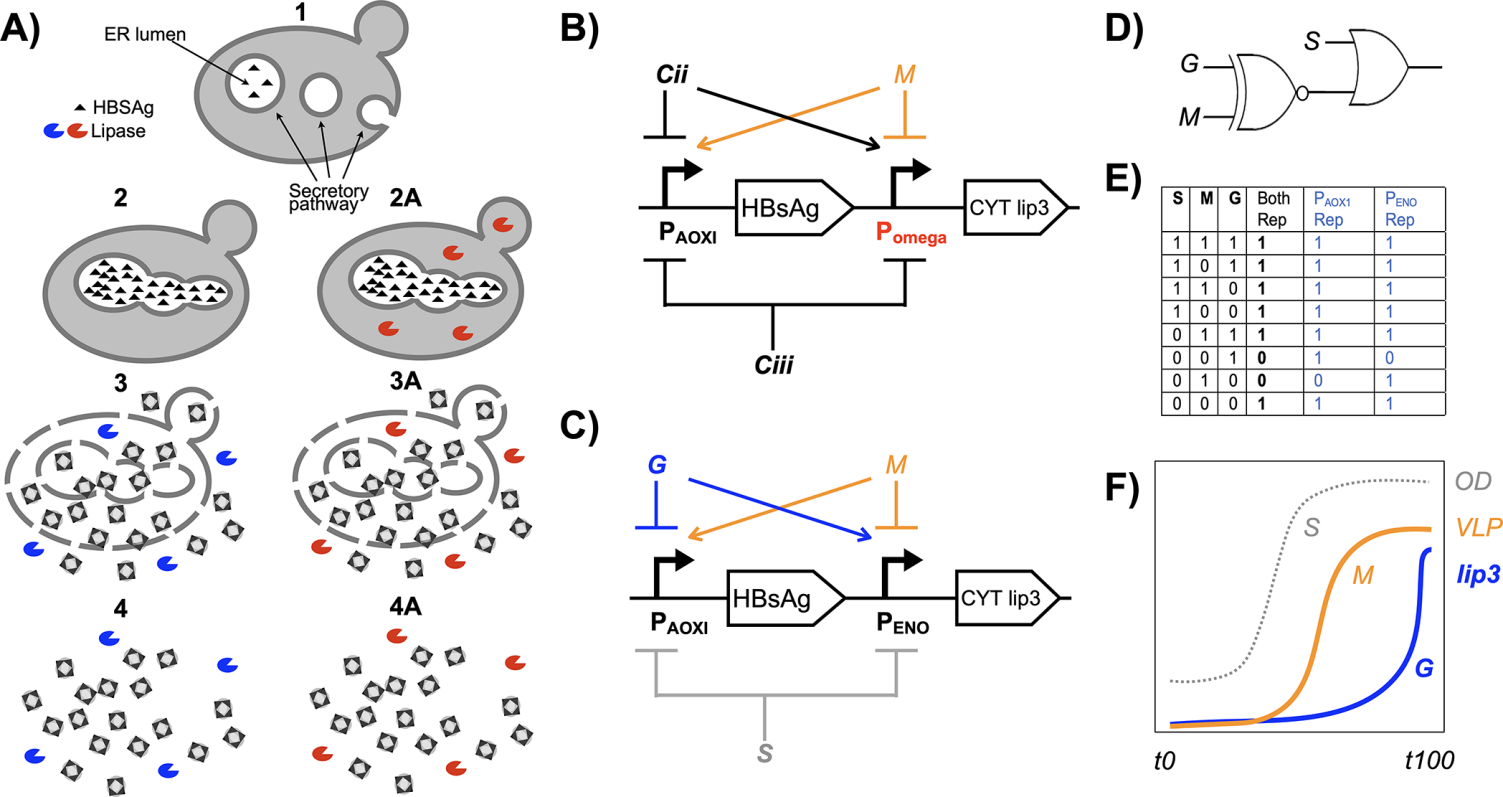
A biological OR(XNOR) logic gate to optimize lipase-assisted bioprocessing of a *K. phaffii* vaccine production strain. (A) Diagram of steps in HBsAg-derived VLP production in *K. phaffii* where (1) recombinant HBsAg protein is translocated to the endoplasmic reticulum (ER) lumen, (2) the ER deforms as HBsAg accumulates, (3) HBsAg forms into VLPs during cell disruption, and (4) addition of an exogenous lipase (blue symbol) for lipid clearance can ease downstream processing.^4^ Steps 2A to 4A show predicted steps where cytosolic lipase (red symbol) is expressed from a transgene within the HBsAg producer cells. A 3-input OR(XNOR) logic gate gives a TRUE output for all input combinations except where only the second or only the third input is true. To implement a biological OR(XNOR) logic gate in *Pichia pastoris*, where each of the three inputs is a carbon source, would require a promoter, other than P_AOXI_, that is repressed by two of the carbon sources and activated by the third; we termed this the “omega” promoter, P_omega_. (B) Diagram of the predicted biological components required for the biological OR(XNOR) logic gate: methanol (M) to repress a notional P_omega_ promoter and induce the P_AOXI_ promoter, a Cii carbon source to repress P_AOXI_ and induce P_omega_, and a Ciii carbon source to repress P_AOXI_ and P_omega_. (C) Design choices: P_ENO_ to provide P_omega_ function, glycerol as Cii carbon source and sorbitol as Ciii to provide relative repression of P_AOXI_ and P_omega_. (D) OR(XNOR) logic gate diagram. (E) OR(XNOR) logic gate truth table. (F) Sketch of a desired pattern of VLP and lipase coexpression and biomass accumulation.

A recombinant *K. phaffii* strain that could co-express the *C. rugosa* Lip3 lipase,^6^ alongside VLPs could potentially achieve the same downstream processing benefits but without the need to add lipase in an exogenous manner (Figure 1A). The two most commonly used promoters for transgene expression in *K. phaffii* are the constitutive P_GAP_ promoter and the strong, methanol-inducible P_AOX1_ promoter. Optimal co-expression control for HBsAg and Lip3 would maximize initial trophophasic cell growth and then HBsAg production over the duration of a bioreactor-based cultivation period and restrict Lip3 expression to a short period at the end of cultivation, after the majority of HBsAg had been accumulated. Using P_GAP_ to direct HBsAg expression^7^ and P_AOX1_ for Lip3 expression would likely result in too little HBsAg production compared to standard processes where P_AOX1_ is used to direct HBsAg expression.^8^ Using P_GAP_ for Lip3 expression and P_AOX1_ for HBsAg expression would direct continuous Lip3 production, exerting a metabolic burden throughout cultivation.

Previously, 2-input biological XNOR logic gates, which compute TRUE only when both inputs are true or both inputs are false, have been designed, built, and tested in *Escherichia coli* with tetracycline and arabinose as the two inputs.^9^ A 3-input OR(XNOR) logic gate computes FALSE when only the second or only the third input is true and TRUE for all other input combinations (Figure 1E).

To address the limitations of conventional HBsAg and Lip3 co-expression, we proposed a 3-input OR(XNOR) biological logic gate in which three carbon sources are inputs, one carbon source capable of supporting trophophasic cell growth and each of the other two being able to induce separate transgene expression (Figure 1B–F). This logic gate would be deployed with P_AOX1_ to direct methanol-inducible HBsAg expression and a second notional promoter to direct Lip3 expression, which for brevity we refer to as the “omega” promoter (P_omega_). Methanol would induce P_AOX1_ and repress P_omega_, a second carbon source, Cii, would induce P_omega_ and repress P_AOX1_, while a third carbon source, Ciii, would support trophophasic cell growth but not have induction activity of methanol or Cii and therefore represent relative inhibition of P_omega_ and P_AOX1_ (Figure 1B).

In previous shake flask studies, methanol induction of P_ENO1_ resulted in 50% less GFP than methanol induction of P_AOX1_,^10^ and the P_ENO1_ promoter was induced by glycerol and repressed by glucose, methanol, and ethanol.^11^ As such we used P_ENO1_ for the P_omega_ function within the OR(XNOR) gate and glycerol as the Cii carbon source as it represses the P_AOX1_, even in the presence of methanol.^12^ Sorbitol is readily utilized by *K. phaffii* as a carbon source but does not induce the P_AOX1_ promoter.^13^ We could locate no direct reports on the effect of sorbitol on the P_ENO1_ promoter so decided to trial it as the Ciii carbon source.

Plasmids pJ9Sag, encoding HBsAg, and pJ9SEATLC, encoding HBsAg and cytosol-targeted lip3 lipase, were each used to stably transform cells of *K. phaffii* strain BG-10 (ATUM) by homologous recombination at the AOX1 locus for a predicted transgene copy number of one, preservation of the Mut+ phenotype, and introduction of the Zeocin resistance phenotype. The resulting strains (Table 1) were cultivated in complex YPD media, with glucose followed by methanol as principal carbon source as a “standard shake flask cultivation”, to confirm functional lip3 and HBsAg expression in the recombinant strains (Figure 2). Cells of the BG-10 and BASEL10 strains were homogenized and centrifuged (Figure 2A, left arm of flowsheet) to isolate a crude cytosolic fraction. Cytosolic fractions from both strains were subjected to nonreducing polyacrylamide gel electrophoresis followed by gel submersion in the fluorogenic lipase substrate MUB. The gel lane containing cytosolic fraction from the BASEL10 strain exhibited a fluorescent signal associated with a band, whereas no equivalent signal was observed for the unmodified BG-10 strain (Figure 2B), indicating the lipase was biologically active.

**Figure 2:**
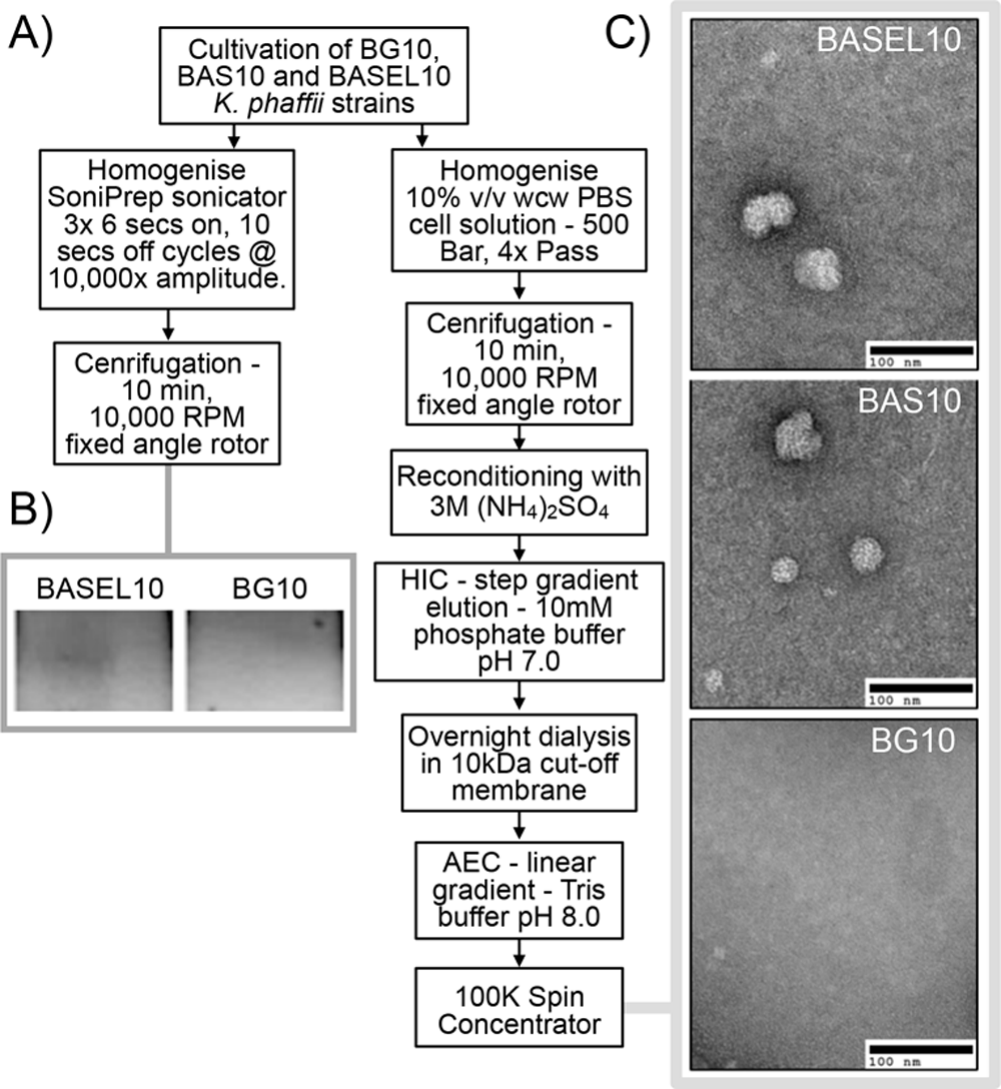
Active co-expression of lipase and HBsAg VLP vaccine in *K. phaffii*. (A) Flowchart of purification steps used to obtain fractions containing V5-tagged CRL lip3 (left arm of chart) and HBsAg-derived VLPs (right arm of chart). Panel B shows cropped images of a zymogram of *in situ* activity of gel bands from strain BASEL10 and BG-10 for conversion of fluorigenic MUB substrate. Panel C shows TEM images obtained for the indicated *K. phaffii* strains.

**Table 1:** Expression Plasmids and Strains in This Study

plasmid	plasmid notes	*Pichia* strain	*Pichia* strain notes
a	a	BG10	parental, IP-free strain from Atum Zeo^S^, Mut+
pJ902-15	*K. phaffii* expression plasmid from ATUM encoding Zeocin resistance (Zeo^R^) gene	a	a
pJ9Sag	expression plasmid encoding HBSAg ORF downstream of P_AOX1_	BAS10	stably transfected with linearized pJ9Sag, Zeo^R^, Mut+
pJ9SEATLC	expression plasmid encoding HBsAg ORF downstream of P_AOX1_ and *C. rugosa* lip3cyt ORF downstream of P_ENO1_	BASEL10	stably transfected with linearized pJ9SEATLC, Zeo^R^, Mut+

a Not applicable.

We next cultivated cells in 50 mL YPD media for VLP purification (Figure 2A, right arm of the flow sheet). For BAS10 and BASEL10 strains, resultant fractions were analyzed by TEM (Figure 2C) to reveal structures consistent with VLPs evidenced in a similar manner by others.^14^ No VLP structures were observed by TEM from equivalent fractions derived from the parental BG10 strain. We inoculated 200 mL YPD media in shake flasks with the BASEL10 strain and changed carbon sources 25 h (t25), 50 h (t50), and 100 h (t100) postinoculation (Figure 3) by pelleting cells then resuspending in YPD media supplemented with the alternate carbon source. As both HBsAg and lip3 lipase are intracellular products, we expected that their abundance would decrease, due to intracellular protein turnover, if their rate of synthesis significantly dropped.

**Figure 3:**
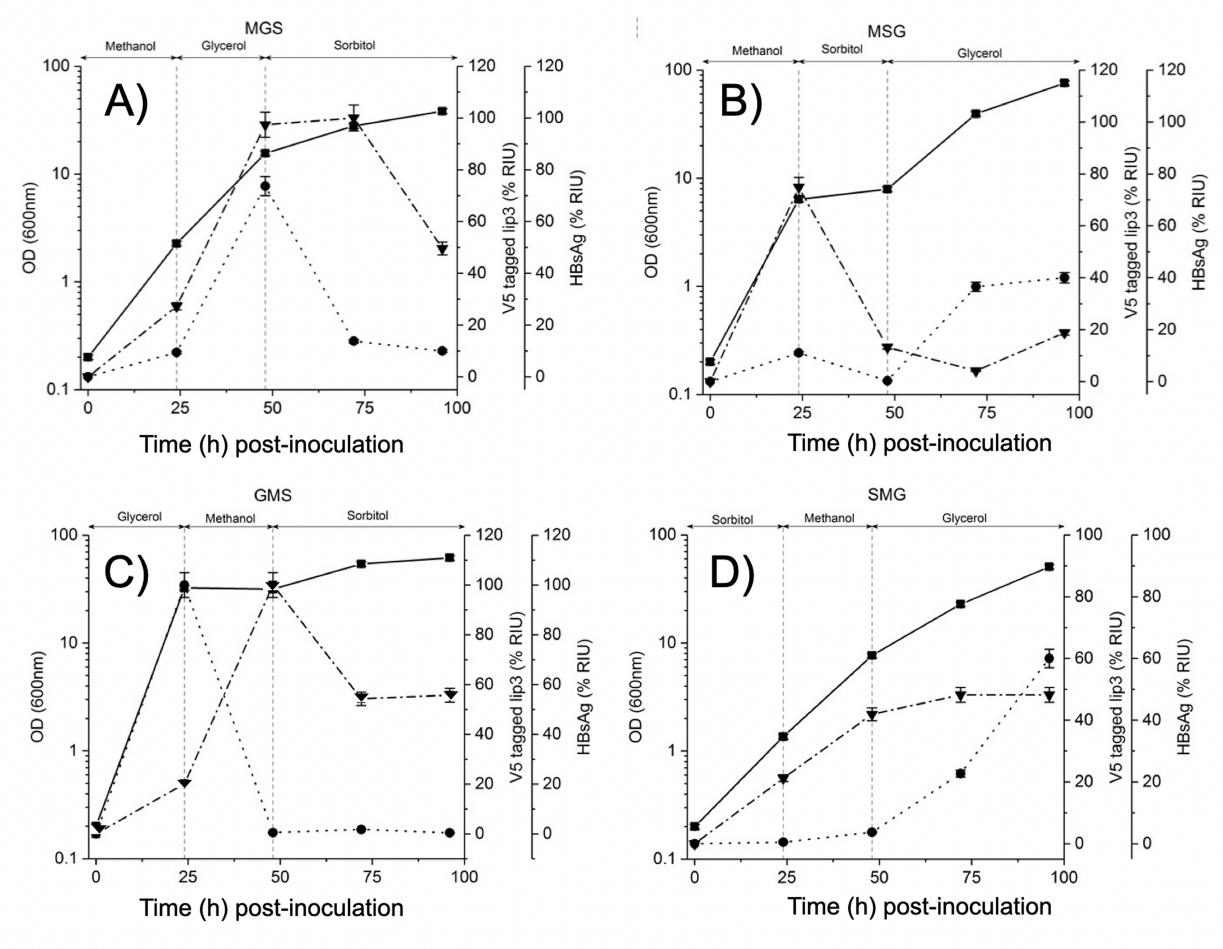
OR(XNOR) gate functions in shake flasks to compute fed carbon source switching inputs. Duplicate (*n* = 2) BASEL10 strain cultivations in 50 mL media in 250 mL baffled shakes over time, with swapped carbon source indicated at the top of each plot. Concentration of HBsAg (inverted triangles) and V5-tagged CRL lip3 (solid circles) was determined by densitometric analysis of Western blot images (see Materials and Methods) and represented as percentage of maximum relative intensity units (% RIU). (A) Carbon source was switched from methanol (M) to glycerol (G) and then to sorbitol (S) at 25 and 50 h postinoculation for all cultivations. (B) Carbon source switching sequence of M to S then G. (C) Carbon source switching sequence of G to M then S. (D) Carbon source switching sequence of S to M then G. Standard error for *n* = 2 was plotted as error bars for each time point.

We first applied the carbon source sequence of methanol followed by glycerol then sorbitol (MGS) (Figure 3A). In the initial presence of methanol only, HBsAg was produced at approximately double the rate of lip3; then upon switching to glycerol HBsAg accumulated at a similar rate to lip3. HBsAg levels only reduced 50 h after the switch away from methanol (75 h postinoculation). We suggest a reservoir of intracellular methanol may have persisted during the cell pelleting procedure to sustain HBsAg induction from 25 to 75 h postinoculation. Switching to sorbitol at t50 resulted in immediate reduction in lip3 and, after a 25-h lag, reduction in HBsAg (Figure 3A).

We next tested OR(XNOR) gate function with methanol followed by sorbitol then glycerol (MSG) (Figure 3B). Initial methanol again induced HBsAg production at a significantly higher rate than lip3. Switching to sorbitol at t25 immediately impacted both HBsAg and lip3 levels, reducing expression of both. Final switching to glycerol at t50 induced lip3 production immediately and HBsAg expression never returned to its t25 level (Figure 3B).

In the initial methanol feed phase of the MGS (Figure 3A) and MSG (Figure 3B) switching regimes, OD varied from ≈2 to ≈7 respectively, while HBsAg varied further, from 0.5% to 6% of maximal levels. Given the relatively low OD levels and the shake flask method of cultivation, we attributed this to expected levels of variability.

With a GMS carbon source switching sequence (Figure 3C), initial glycerol strongly induced lip3 expression. Switching to methanol at t25 resulted in sharp reduction in lip3 levels, which remained low throughout the final 50 h of cultivation during which sorbitol was provided as carbon source. HBsAg production increased after the t25 switch to methanol and reduced after the t50 switch to sorbitol.

Finally, we tested an SMG carbon source switching sequence (Figure 3D). Initial sorbitol and methanol phases resulted in low levels of lip3 production, with lip3 levels only significantly increasing after the switch to glycerol and in the final 25 h of cultivation. Levels of HBsAg production were relatively low during initial sorbitol phase and increased upon the switch to methanol to a level which was maintained for the 50 h after the switch to glycerol.

When cell mass achieved OD 7 or above, a switch from either methanol or glycerol to sorbitol (Figure 3A–C) coincided with a reduction in the level of both lip3 and HBsAg, either immediately or after a lag. This observation is consistent with our expectations of the function of the biological OR(XNOR) gate, with sorbitol causing relative repression of the P_AOXI_ and P_ENOI_ promoters compared to methanol and glycerol, respectively. It remains possible that the presence of sorbitol acts to favor direct intracellular degradation of lip3 and HBsAg proteins, and future investigations will be needed to elucidate that mechanism of sorbitol’s effects in this situation.

The SMG sequence of carbon source switching resulted in the temporal profile of lip3 and HBsAg expression (Figure 3D) that we had anticipated when designing the OR(XNOR) gate (Figure 1F), with expression of the potentially process-enhancing lipase only increasing significantly in the final quarter of the cultivation period, with HBsAg expression being maintained throughout.

We next cultivated the BASEL10 strain in 150 mL yeast nitrogen base (YNB) media in an Eppendorf DasGip 250 mL bioreactor. Monitoring the three carbon sources during bioreactor cultivation (Figure 4A) revealed that sorbitol was consumed at a slow rate by cells throughout cultivation but never fell below 50% of the initial concentration achieved at bolus addition. The two steep dips in sorbitol concentration (Figure 4A) are most likely caused by the approximately 1/5th and 1/6th dilution of the culture upon methanol and glycerol additions. By contrast, approximately 70% of the methanol bolus and over 95% of the glycerol bolus were consumed within 10 h of their addition. DO spikes following depletion of the methanol and glycerol boluses (Figure 4B) indicated that these carbon sources were preferentially metabolized in the presence of sorbitol. Cell biomass (Figure 4B) did not achieve levels typically observed when glycerol is used as the carbon source for trophophase growth in optimized cultivation regimes. However, the profile of lip3 and HBsAg expression (Figure 4C) followed the same overall desired pattern observed previously with the SMG sequence in shake flasks (Figure 3D). It is also notable that small levels of glycerol production were detected at approximately 25 and 50 h postinoculation, during the early phase of cell growth (Figure 3A), along with small concomitant increases in Lip3 expression (Figure 3C). Glycerol biosynthesis^15^ is understood to occur in *Saccharomyces cerevisiae* and *K. phaffii* in response to osmotic stress^16^ transiently experienced by cells due to the changes in their environment.

**Figure 4:**
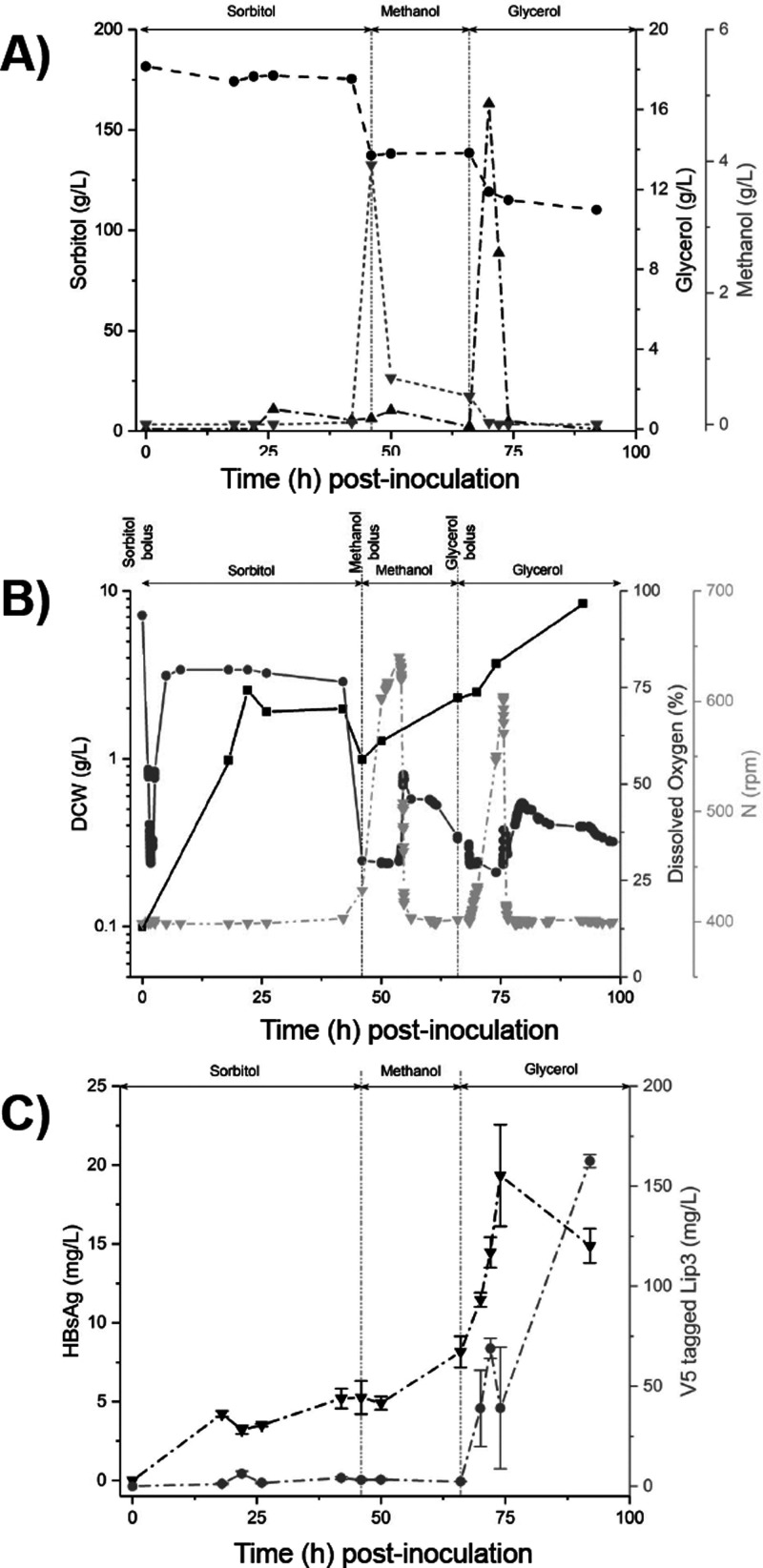
OR(XNOR) biological gate function during cultivation in bioreactor. Plots covering eight metrics arising from BASEL10 strain cultivation in 150 mL media in 0.25 L DasGip bioreactor over time, with carbon source bolus addition indicated at the top of each plot. (A) Impeller revolutions per minute (*N*), dissolved oxygen percentage, and w/v dry cell weight (DCW) in light gray down triangle, gray up triangle, and black circle data points, respectively. (B) Concentration of methanol, glycerol, and sorbitol in light gray down triangle, gray circle, and black square data points, respectively. (C) Concentration of HBsAg and V5-tagged CRL lip3 lipase in light gray circle and gray down triangle data points, respectively. Error bars represents standard deviation around *n* = 2 dot blot band intensity measurements.

Approximately 80% of total HBsAg yield was accumulated prior to increased induction of lipase expression (Figure 4C, 75 h postinoculation). Reported HBsAg yield yields from *K. phaffii* vary depending on production regime, ranging from 12.5 μg/L^17^ to 7 g/L.^8^ Further optimization, of OR(XNOR) gate components and/or bioreactor cultivation regime will be needed to increase the ≈20 mg/L HBsAg yield observed in Figure 4C, 75 h postinoculation.

The data presented here proves that a given configuration of promoter and carbon source choices can act together to provide a sophisticated logic gate capability in a biotechnology workhorse yeast such as *K. phaffii* (*P. pastoris*) utilized in industrially robust and scalable settings.

## Materials and Methods

All chemicals were purchased from Sigma Chemical Co. Ltd. (Dorset, UK), unless stated otherwise.

### Plasmid Design and Assembly

Eurogentec (Belgium) performed all DNA synthesis and assembly. The plasmid pJ9Sag was designed and assembled based on the pJ902-15 (ATUM, California, USA) *K. phaffii* backbone and encoded the HBsAg (GenBank accession number J02205.1) open reading frame (ORF) downstream of the P_AOX1_ promoter and upstream of the AOX1 gene transcription terminator (TT). pJ9SEATLC encoded this same HBsAg expression cassette plus a further downstream gene featuring an ORF encoding the *C. rugosa* Lip3 lipase (GenBank accession number X66006.1) modified so that its native amino-terminal (N-terminal) secretion signal^18^ was replaced with an N-terminal V5 epitope tag downstream of the P_ENO1_ promoter and upstream of the AOX1 TT.

### Generating Recombinant *K. phaffii* Strains

Plasmids pJ9SEATLC and pJ9Sag were each linearized at a lone *Sac*I site in the P_AOX1_ promoter, then used to transform ATUM *K. phaffii* strain BG10 cells by electroporation and integration at the P_AOX1_ promoter locus within recipient cells. This generated the recombinant strains BAS10 and BASEL10, harboring genomically integrated pJ9Sag and pJ9SEATLC respectively. Both strains were propagated using YPD agar plates (1% w/v yeast extract and 2% w/v peptone, dextrose, and agar) containing 1 mg/mL Zeocin to select for Zeocin-resistance. Colony PCR with primers GGTAAGCCTATCCCTAAC and TTACACAAAGAACAGCAG confirmed the presence of the *C. rugosa* Lip3 ORF and ATGGAGAACATCACATCA and AAATGTATACCCAGAGAC confirmed the presence of the HBsAg ORF. For both strains, we inferred that at least one insertion event occurred at the intended locus because the *Sac*I site used for plasmid linearization truncates the P_AOXI_ promoter, so the phenotype of methanol-based induction of HBsAg expression (Figure 3) could only manifest if the genomic insertion re-formed a completed copy of the P_AOXI_ promoter, as intended.

### Standard Shake Flask Cultivation

Recombinant *K. phaffii* strains were cultivated in 50 mL YPD liquid media in 250 mL shake flasks at 30 °C under 250 rpm shaking overnight prior to the zymogram experiment reported in Figure 2B. Samples used for Western blotting and the transmission electron microscopy (TEM) data in Figure 2C were grown in 200 mL YPD media in 2 L baffled shake flasks for 48 h. Cells were then pelleted and resuspended in 20 mM succinate buffer containing 0.7% v/v methanol and maintained in this induction solution at 30 °C under 250 rpm shaking for another 48 h with daily addition of methanol to 0.7% v/v.

### Purification of VLPs from Standard Shake Flask Cultivation

After 48 h of methanol induction, cells were resuspended in phosphate buffered saline (PBS) at 10% v/v wet cell weight (wcw), homogenized via five 500 bar passes through a Gaulin lab40 homogenizer, then centrifuged with an Eppendorf 5804R centrifuge fitted with an FA-45-6-30 rotor at 10000*g* for 10 min at room temperature. The supernatant was then reconditioned using 3 M (NH_4_)_2_SO_4_ to a final concentration of 0.6 M (NH_4_)_2_SO_4_ and loaded onto a pre-equilibrated 5 mL HiTrap Butyl-S 6 column (GE Healthcare) at pH 7.0 for hydrophobic interaction chromatography (HIC).

A step gradient was used to elute the HBsAg from the column. The eluted sample was dialyzed using 10 kDa MWCO Slide-A-Lyzer Dialysis Cassettes (Thermo Scientific) in 20 mM Tris pH 8.0 buffer. The dialyzed sample was then loaded on to the 5 mL HiTrap Q Sepharose Fast Flow (GE Healthcare) anion exchanger column for further purification. An elution buffer of 25 mM Tris-HCl, 0.5 M NaCl was used to clean the column. HBsAg was eluted with linear gradient of 20 column volumes with buffer containing 1 M NaCl. The eluted fraction was then concentrated, and buffer was exchanged into 5 mM 4-(2-hydroxyethyl)-1-piperazineethanesulfonic acid (HEPES) using a 10 kDa MWCO spin concentrator. This purified sample in HEPES buffer was then used for transmission electron microscopy (Figure 2A,C).

### Western Blotting of Samples from Standard Shake Flask Cultivation

Four milliliters of *K. phaffii* inoculum from standard shake flask cultivation was centrifuged at 10000*g* for 5 min, supernatant was removed, and cell pellet was resuspended in 0.5 mL of the disruption buffer^19^ consisting of 50 mM 3-(*N*-morpholino)propanesulfonic acid (MOPS), 1 mM 4-benzenesulfonyl fluoride hydrochloride, 5 mM dithiothreitol, 5 mM ethylenediaminetetraacetic acid, and 0.1% (v/v) Triton X-100, adjusted to pH 7.5 using 1 M sodium hydroxide. The resulting cell solution was disrupted using a SoniPrep sonicator with 6 s on and 10 s off for 3 cycles with 10000× amplitude. The sonicated samples were subjected to SDS PAGE and membrane transfer using standard Western blotting methods. For HBsAg detection, the membrane was transferred to a 1:1000 dilution of a horseradish peroxidase (HRP)-linked polyclonal anti-HBsAg rabbit primary antibody (Abcam, Cat No. ab20878) in Tris-buffered saline (TBS) buffer containing 0.1% v/v Tween 20 (TBST) buffer, with shaking at 2–8 °C overnight before washing and visualization. For *C. rugosa* Lip3 lipase detection, a 1:1000 dilution of a monoclonal anti-SV5-Pk1 primary antibody (Abcam, Cat. No. ab27671) in TBST buffer was used, with shaking at 2–8 °C overnight. The membrane was then washed three times with TBST for 5 min each, followed by a 15 min wash in TBS buffer. The membrane was then incubated with a 1:1000 dilution of a goat anti-mouse IgG (Alexa Fluor 488) secondary antibody from Abcam (Cat No. ab150113) in 2.5% w/v skimmed milk TBST. Membranes were then washed, and antibody was detected using standard ECL substrate and procedures (Bio-Rad) and an Amersham Imager 600, with bands quantified using ImageJ and Origin (OriginLab, Northampton, MA, USA). Membranes were spotted with 25 ng of recombinant HBsAg (Cat. No. 5015.005, Aldevron, Madison, WI, USA) or 2.5 ng V5 peptide (Abcam, Cat No. ab15829) as standard for quantification.

### Transmission Electron Microscopy (TEM) of Purified VLPs from Standard Shake Flask Cultivation

Purified HBsAg samples were concentrated using a 0.5 mL Amicon Ultra-0.5 Centrifugal Filter Unit with Ultracel-10 membrane and 10 kDa MWCO (Amicon, Cat No. UFC501096) concentrator and were buffer-exchanged with pH 7.5, 5 mM HEPES buffer. The samples were negatively stained using a 400-mesh copper carbon coated grid held by forceps. Two microliters of sample was applied onto the grid surface and, after 2–3 min, drawn off from the edge of the grid with Whatman filter paper. Care was taken to ensure the grid did not completely dry before being placed sample-side down onto a drop of pH 7.3, 20 mM phosphate buffer for 10 s. Excess buffer was also drawn off from the edge of the grid with Whatman filter paper. The grid was stained immediately by placing it onto a drop of 1% v/v uranyl acetate in distilled water for 30 s. Excess stain was then drawn off the grid, which was then air-dried for several minutes before observation. The grid was then imaged using a Jeol1010 TEM (JEOL Ltd., Japan), and images were recorded using a Gatan Orius (Gatan Ametek, USA) camera at 180000× magnification. The microscope used for the images in Figure 2C was a Phillips CM12 (FEI UK Ltd.) with an ATM camera system used for image capture.

### Zymogram Analysis of Homogenates from Standard Shake Flask Cultivation

Cell samples were centrifuged, and cell pellet was resuspended in 0.5 mL of 25 mM Tris-HCl, pH 7.5. The resulting cell solution was disrupted using a SoniPrep sonicator with 6 s on and 10 s off for 3 cycles with 10000× amplitude. Sonicates were centrifuged at 10000*g* for 10 min at room temperature and 20 μL of supernatant was run on a 4–16% BisTris SDS PAGE gel under nonreducing conditions. In common with methods reported by others,^20^ the postelectrophoresis gel was soaked in 2.5% v/v Triton X-100 for 45 min at room temperature on an orbital shaker. This was followed by two 30 min washes with pH 7.5, 25 mM Tris-HCl. A lipase substrate, 2.5 mM 4-methylumbelliferyl butyrate (MUB), dissolved in 50% v/v methanol and pH 7.5, 25 mM Tris-HCl buffer, was spread onto the gel and incubated at room temperature on an orbital shaker for 60 min. Gel images were captured using an Amersham Imager 600 device.

### Cultivation with Carbon Source Switching

BASEL10 strain cells were grown in 200 mL YPD media in 2 L baffled shake flasks. Cells were pelleted every 24 h by centrifugation at 5000*g* for 5 min at room temperature. The supernatant media was discarded; the cell pellet was washed once with PBS and then resuspended in the same volume of fresh media supplemented with either 1% v/v glycerol, 1 M sorbitol, or 1% v/v methanol, depending on the sequence of carbon source switching.

BASEL10 strain cells were also cultivated in 250 mL YNB media in 2 L baffled shake flasks until an OD_600nm_ of 20 was achieved. Seven milliliters of this inoculum was used to inoculate 150 mL YNB with 2 M sorbitol in an Eppendorf DasGip 0.25 L bioreactor. A dissolved oxygen (DO) cascade was employed to maintain a minimum 30% DO with an agitation rate that ranged from 400 to 6500 rpm. Solutions of 17.5% w/v ammonia and 14% v/v orthophosphoric acid were used to maintain pH at 6.0. The culture was maintained at a temperature of 30 °C. At 46 h postinoculation, a bolus of methanol was added in the form of 50 mL of 5% v/v methanol in YNB medium. At 66 h postinoculation, a bolus of glycerol was added in the form of 50 mL of 10% v/v glycerol in YNB medium.

### Dot Plot Immunoquantification of Samples from Cultivation with Carbon-Source Switching

Samples were prepared in the same manner as for Western blotting up to the step prior to SDS PAGE. At this step, instead of SDS PAGE, samples were serially diluted prior to being loaded directly onto a nitrocellulose membrane (Thermo Scientific) by pipetting 1 μL aliquots in a regular grid pattern of dots. The membrane was then processed as for Western blotting, probing for Lip3 or HBsAg. The intensity of the resultant signal from the dots of antigen was measured using an Amersham 600 gel imager and analyzed using the Origin fitting algorithm (OriginLab, Northampton, MA).

### HPLC Measurement of Carbon Source

During bioreactor cultivation, levels of sorbitol, glycerol, and methanol were determined using a Dionex HPLC system (Camberley, UK) with a 300 × 7.8 mm^2^ Bio-Rad Aminex HPX-87H reverse phase column (Bio-Rad Laboratories, Richmond, CA, USA). Chromeleon client 6.60 software was used for signal separation and analysis. Standard curves were prepared using the carbon source bolus solutions.

## References

[ref1] Xia P. F.; Ling H.; Foo J. L.; Chang M. W. Synthetic genetic circuits for programmable biological functionalities. Biotechnol Adv. 2019, 37 (6), 107393 10.1016/j.biotechadv.2019.04.015. 31051208

[ref2] Duman-Özdamar Z. E.; Binay B. Production of Industrial Enzymes via Pichia pastoris as a Cell Factory in Bioreactor: Current Status and Future Aspects. Protein J. 2021, 40 (3), 367–376. 10.1007/s10930-021-09968-7. 33587243

[ref3] Valenzuela P.; Medina A.; Rutter W. J.; Ammerer G.; Hall B. D. Synthesis and assembly of hepatitis B virus surface antigen particles in yeast. Nature 1982, 298, 347–50. 10.1038/298347a0. 7045698

[ref4] Jin J.; Chhatre S.; Titchener-Hooker N. J.; Bracewell D. G. Evaluation of the impact of lipid fouling during the chromatographic purification of virus-like particles from Saccharomyces cerevisiae. J. Chem. Technol. Biotechnol. 2010, 85, 209–215. 10.1002/jctb.2290.

[ref5] Bandyopadhyay S. K.; Morris S. A.; Nesbeth D. N.; Bracewell D. G. Lipid reduction to improve clarification and filterability during primary recovery of intracellular products in yeast lysates using exogenous lipase. J. Chem. Technol. Biotechnol. 2021, 96, 3166–3174. 10.1002/jctb.6871.

[ref6] Pernas M.; López C.; Prada A.; Hermoso J.; Rúa M. L. Structural basis for the kinetics of Candida rugosa Lip1 and Lip3 isoenzymes. Colloids Surfaces B Biointerfaces 2002, 26, 67–74. 10.1016/S0927-7765(01)00309-5.

[ref7] Vassileva A.; Chugh D. A.; Swaminathan S.; Khanna N. Expression of hepatitis B surface antigen in the methylotrophic yeast Pichia pastoris using the GAP promoter. J. Biotechnol. 2001, 88, 21–35. 10.1016/S0168-1656(01)00254-1. 11377762

[ref8] Gurramkonda C.; Adnan A.; Gäbel T.; Lünsdorf H.; Ross A.; Nemani S. K.; Swaminathan S.; Khanna N.; Rinas U. Simple high-cell density fed-batch technique for high-level recombinant protein production with Pichia pastoris: Application to intracellular production of Hepatitis B surface antigen. Microb. Cell Fact. 2009, 8, 13 10.1186/1475-2859-8-13. 19208244 PMC2646686

[ref9] Bonnet J.; Yin P.; Ortiz M. E.; Subsoontorn P.; Endy D. Amplifying genetic logic gates. Science 2013, 340 (6132), 599–603. 10.1126/science.1232758. 23539178

[ref10] Stadlmayr G.; Mecklenbräuker A.; Rothmüller M.; Maurer M.; Sauer M.; Mattanovich D.; Gasser B. Identification and characterisation of novel Pichia pastoris promoters for heterologous protein production. J. Biotechnol. 2010, 150, 519–529. 10.1016/j.jbiotec.2010.09.957. 20933554

[ref11] Cregg J.; Tolstorukov I. *P. pastoris* ADH promoter and use thereof to direct expression of proteins. United States patent US8,222,386, 2012.

[ref12] Koutz P.; Davis G. R.; Stillman C.; Barringer K.; Cregg J.; Thill G. Structural comparison of the *Pichia pastoris* alcohol oxidase genes. Yeast 1989, 5, 167–177. 10.1002/yea.320050306. 2660463

[ref13] Carly F.; Niu H.; Delvigne F.; Fickers P. Influence of methanol/sorbitol co-feeding rate on pAOX1 induction in a *Pichia pastoris* Mut+ strain in bioreactor with limited oxygen transfer rate. J. Ind. Microbiol. Biotechnol. 2016, 43, 517–523. 10.1007/s10295-015-1722-6. 26790417

[ref14] Hosseini S. N.; Sarvari T.; Bashiri G.; Khatami M.; Shojaosadati S. A. Assessing virus like particles formation and r-HBsAg aggregation during large scale production of recombinant hepatitis B surface antigen from Pichia pastoris. Int. J. Biol. Macromol. 2019, 139, 697–711. 10.1016/j.ijbiomac.2019.08.019. 31381908

[ref15] Näätsaari L.; Mistlberger B.; Ruth C.; Hajek T.; Hartner F. S.; Glieder A. Deletion of the Pichia pastoris KU70 homologue facilitates platform strain generation for gene expression and synthetic biology. PLoS One 2012, 7 (6), e39720 10.1371/journal.pone.0039720. 22768112 PMC3387205

[ref16] Hohmann S. Osmotic stress signaling and osmoadaptation in yeasts. Microbiol Mol. Biol. Rev. 2002, 66 (2), 300–72. 10.1128/MMBR.66.2.300-372.2002. 12040128 PMC120784

[ref17] Bardiya N. Expression in and purification of Hepatitis Bsur- face antigen (S-protein) from methylotrophic yeast Pichia pastoris. Anaerobe 2006, 12, 194–203. 10.1016/j.anaerobe.2006.05.002. 16931065

[ref18] Uemura H.; Jigami Y.; Tanaka H.; Toshimitsu N.; Paterson M.; Nakasato S. Nucleotide sequence of the 5′ flanking region responsible for the enhancement of the expression of yeast enolase 1 gene. J. Biochem. 1985, 98, 859–62. 10.1093/oxfordjournals.jbchem.a135345. 3003042

[ref19] Bláha B. A. F.; Morris S. A.; Ogonah O. W.; Maucourant S.; Crescente V.; Rosenberg W.; Mukhopadhyay T. K. Development of a high-throughput microscale cell disruption platform for Pichia pastoris in rapid bioprocess design. Biotechnol. Prog. 2018, 34, 130 10.1002/btpr.2555. 28884522

[ref20] Singh R.; Gupta N.; Goswami V. K.; Gupta R. A simple activity staining protocol for lipases and esterases. Appl. Microbiol. Biotechnol. 2006, 70, 679–682. 10.1007/s00253-005-0138-z. 16170531

